# Prevalence of human papillomavirus and subtype distribution in male partners of women with cervical intraepithelial neoplasia (CIN): a systematic review

**DOI:** 10.1186/s12879-019-3805-x

**Published:** 2019-02-26

**Authors:** Anargyros Skoulakis, Serafim Fountas, Myrto Mantzana-Peteinelli, Kleoniki Pantelidi, Efthymia Petinaki

**Affiliations:** Department of Microbiology, Medical School, University of Thessaly, Larissa, Greece

**Keywords:** HPV, Male partners, CIN, Systematic review

## Abstract

**Background:**

Human Papillomavirus (HPV) infection is estimated to be the most common sexually transmitted infection. The present systematic review summarizes data regarding the prevalence of HPV and the distribution of subtypes in heterosexual male partners of women, who were diagnosed with any grade of cervical intraepithelial neoplasia (CIN).

**Methods:**

We conducted a systematic review of the literature by Medline and Google Scholar databases using the terms “Human Papillomavirus” or “HPV” plus “men” or “male partners” or “women with CIN”. We included original published English-language articles published from 1/1/2000 until 1/1/2018 that had screened male partners of women with CIN using HPV DNA testing. We excluded studies that they overlapped with other included studies or were unrelated to the study subject.

**Results:**

We included a total of 12 publications, which reported the prevalence of HPV in free-clinical signs male partners of women with CIN. The largest proportion of the studies were from South America (seven studies), and the rest from Europe. The mean age of participants was 35.18 + − 3.47 years. HPV prevalence ranged from 12.9 to 86%; the total HPV prevalence among the studies was 49.1%, while ten out twelve studies (83.3%) demonstrated prevalence > 20%. Between the studies, the distribution of HPV subtypes varied on the basis of the method used, on the population and on the geographic region. A great variety of subtypes were detected, including 6, 11, 16, 18, 31, 33, 40, 42, 45, 51, 52, 53, 54, 56, 57, 58, 59, 61, 62, 66, 68, 81 and 83. In six studies the HPV 16 was the most frequent, while in two others the HPV 6 and HPV 83.

**Conclusions:**

Until now, there are not precise screening or surveillance guidelines for the management of partners of women with CIN. This population is frequently colonized by various HPV subtypes and therefore need to be screened in an effort to reduce the infection in both sexes. The screening test could include detection/identification of HPV subtypes by a molecular assay, followed by peniscopy only in the positive cases.

## Background

Human Papillomavirus infection (HPV) is estimated to be among the most common sexual transmitted infections. Most HPV infections are asymptomatic or subclinical and become undetectable over time. It is well known that some HPV subtypes can cause anogenital warts, dysplastic and/or neoplastic lesions in women and men (heterosexual and men who have sex with men), generating a considerable economic distress within societies [1–3].

There are more than 150 HPV subtypes, which have been grouped according to their oncogenic capacity into High-Risk (HR) and Low-Risk (LR) [4]. Epidemiological studies show that HR are associated in women with invasive cervical cancer and its precursor lesion, the cervical intraepithelial neoplasia (CIN), whereas in men HR subtypes can cause head and neck squamous carcinoma and penile cancer [5, 6].

CIN is a potentially premalignant transformation and dysplasia of squamous cells of the cervix, caused mainly by the HR-HPV types 16 and 18 [7]. Usually, CIN is eliminated by the host’s immune system without any intervention, but, in some cases, when it left untreated, CIN can progress to cervical cancer [8]. Even though there is a vast bibliography regarding the management of the women diagnosed with CIN, there is very limited number of studies focused on the measures that must be applied in male sexual partners of women with CIN. A positive result for HPV infection usually stress women, who are worried about disclosing the result to others and the fact that there is not a clear management of their sexual partners make the disclosing even more difficult [9]. Previous studies have demonstrated that partners of women with CIN can be infected by the virus, while, the risk of developing cancer seems to be higher in men’s second wives when their first wives died from cervical cancer [10].

The present study summarizes data regarding the global prevalence of HPV and the distribution of subtypes in heterosexual male partners of women, who were diagnosed with any grade of CIN. The scope of this review primarily focuses on the characteristics and the results of the selected studies. Assessing the prevalence of HPV infection of male partners is the first step in order to understand the natural history of HPV in couples with women with CIN, and to finally clarify the management of the male partners.

## Methods

In this systematic review we conducted a systematic search in two online databases, Medline and Google Scholar, searching for studies published from 1/1/2000 until 1/1/2018. For our research we use the terms “HPV” OR “Human Papillomavirus”, plus “men”, “male partners” and “women with CIN”. References cited in retrieved articles were also assessed. Eligible studies had to: 1) screen a population of heterosexual male partners of women with CIN, 2) include HPV DNA testing, and 3) be written in English language; all of which were including criteria. The evaluation of articles was performed based on their relevance of the title, abstract and manuscript review. In order to minimize the risk of bias, the evaluation of the articles was performed by two reviewers, independently. We performed a qualitative synthesis of the data for the prevalence and subtype distribution, since the articles varied significantly based on the study design, on the participants characteristics and on the molecular assays used. The prevalence of HPV in the selected studies was calculated by dividing the number of HPV-positive male partners by the total number of male partners of women with CIN. Unfortunately, there were no data for male partners of women without CIN in order to conclude if male partners of women with CIN have higher risk for HPV infection. Hence, no further statistical analysis was possible to be done. All the computations were calculated by R program (RStudio Team (2015). RStudio: Integrated Development for R. RStudio, Inc., Boston, MA URL http://www.rstudio.com/).

## Results

From the 7682 abstracts reviewed, 35 articles were selected and among them, 12 met the inclusion criteria (Table 1) [11–22]. Reasons for the exclusion of 23 articles were that they overlapped with other included studies, they were written in non-English language or they were unrelated to the study subject (see Table 2).

**Table 1:** Characteristics of studies included

Name, year	Time of samples collection	Type of study	Region	Inclusion criteria of the studies included	Exclusion criteria of the studies included
Bleeker 2005	1995–2002	Case- control study	Netherlands	Regular male sexual partners of women with CIN and men visiting the outpatient non-STD clinic	Men with a sexually transmitted disease or with anogenital cancer
Rosenblatt 2004	1999–2001	Case- control study	Brazil	Partners of women having CIN and partners of women without CIN	None
Rombaldi 2006	2003–2004	Cross- sectional study	Brazil	Male sexual partners of women with CIN	None
Giraldo 2008	2003–2005	Cross- sectional study	Brazil	Asymptomatic men who were the sexual partners of women who had a histopathological diagnosis of any low-grade squamous intraepithelial lesions (LSIL)	Sexual partners of women with high-grade lesions
Benevolo 2008	2004–2006	Cross- sectional study	Italy	Italian clinically healthy men, monogamous sexual partners of women affected previously or presently by cervical intraepithelial neoplasia (CIN1 to CIN3) and /or with a positive result of HPV DNA.	Circumcised men and men who reported any previous episode of a sexually transmitted disease. Use of condoms the last 12 months
Guzman-Esquivel 2009	2004–2005	Case- control study	Mexico	Stable male sexual partners, of women with CIN and male sexual partners of women with normal cervical uterine cytology	Men presenting with penile or genital alterations such as genital herpes pediculosis blenorragia and psoriasis and men who were HIV-positive, receiving antiviral or immuno-modulating treatment and men who had received radiotherapy or chemotherapy. Men whose samples were insufficient or inadequate for DNA extraction or if there had been technical errors during their processing.
Martin-Ezquerra 2012	2006–2007	Cross- sectional study	Spain	Heterosexual male partners of women, who had been diagnosed with a CIN II or III during the 6 months prior to enrollment	Partners of women with pregnancy and any kind immune-suppression
Afonso 2013	2000–2010	Cross- sectional study	Brazil	Female patients presenting CIN as well as their male sexual partners (Group I) and asymptomatic couples (Group II)	None
de Lima Rocha 2012	N/A	Cross- sectional study	Brazil	Stable male partners (for at least 6 months) of women with cytological or histopathological diagnosis of cervical squamous intraepithelial lesions associated to HPV infection.	None
Rob 2017	2013–2015	Cross- sectional study	Czech republic	Monogamous male partners of women with histologically verified CIN (grades II-III) or genital warts	Length of the current relationship, intercourse with other sexual partners and HPV vaccination
Vargas 2016	2015 (3 months)	Cross- sectional study	Colombia	Women engaged in a regular relationship and presenting CIN and their sexual partners	None
Lopez-Diez 2017	2013–2015	Cross- sectional study	Spain	Asymptomatic men, more than 18 years old, not vaccinated against HPV, whose sexual partners (regular sexual intercourse more than 1 year) had presented high grade squamous cervical lesions (CIN II or CIN III in the previous 6 months)	None

**Table 2:** First Name, Year, Title, Journal and Reasons for Exclusion of 23 studies

Name, Year	Title of article	Journal	Reasons for exclusion
Pan LJ et al.; 2018	HPV infection of the external genitalia in men whose female partners have cervical HPV infection	Zhonghua Nan Ke Xue. 24:516–9	Article in Chinese
Marcellusi A et al., 2015	Health utilities lost and risk factors associated with HPV-induced diseases in men and women: the HPV Italian collaborative study group	CLin Ther 37:156–167	Unrelated to the study subject
Drabina J et al., 2015	Prevalence of HPV DNA among male sexual partners of women diagnosed with CIN and early invasive cervical cancer	Przegl Lek, 72: 611–5	Article in Polish
Lorenzon L et al., 2014	Prevalence of HPV infection among clinically healthy Italian males and genotype concordance between stable sexual partner	J Clin Virol, 60:264–9	Overlap with a previous study by the same group, which was included (Benevolo et al. 2008). In addition, the men were stable partners of women who had been HPV/CIN positive in the past 3 years, but whose pathological data at enrolment were not available so it was not possible to distinguish the participants with HPV + partners from participants with CiN partners.
Carestiato FN et al., 2006	Prevalence of human papillomavirus infection in the genital tract determined by hybrid capture assay	Braz J Infect Dis. 10:331–6.	Unrelated to the study subject
Varela JA et al.; 2006	Research on sexually transmitted infections in asymptomatic heterosexual males whose partners have cervical intraepithelial neoplasia	Actas Dermosifiliogr 97:319–22.	No HPV detection; article in Spanish
Bleeker MC et al., 2005	HPV type concordance in sexual couples determines the effect of condoms on regression of flat penile lesions	Br J Cancer 92: 1388–92	Overlap with a study from the same research group that was included (Bleeker 2005)
Hogewoning CJ et al., 2003	Condom use promotes regression of cervical intraepithelial neoplasia and clearance of human papillomavirus: a randomized clinical trial.	Int J Cancer. 107: 811–6	Unrelated to the study subject
Bleeker MC et al., 2003	Condom use promotes regression of human papillomavirus-associated penile lesions in male sexual partners of women with cervical intraepithelial neoplasia	Int J Cancer. 107:804–10.	Overlap with a study from the same research group that was included (Bleeker 2005)
Finan RR et al., 2002	Identification of Chlamydia trachomatis DNA in human papillomavirus (HPV) positive women with normal and abnormal cytology.	Arch Gynecol Obstet. 266:168–71	Unrelated to the study subject
Bleeker MC et al., 2002	Penile lesions and human papillomavirus in male sexual partners of women with cervical intraepithelial neoplasia	J Am Acad Dermatol. 47:351–7	Overlap with a study from the same research group that was included (Bleeker 2005)
Tamim H et al., 2002	Cervicovaginal co-infections with human papillomavirus and Chlamydia trachomatis	Diagn Microbiol Infect Dis.43: 277–81	Unrelated to the study subject
Bleeker MC et al., 2006	Flat penile lesions: the infectious “invisible” link in the transmission of human papillomavirus	Int J Cancer. 119:2505–12.	Overlap with the study by Bleeker MC et al., 2002
Franceschi S et al., 2002	Prevalence and determinants of human papillomavirus genital infections in men	Br J Cancer 86:705–11	Combined data collected in five case-control studies of invasive cervical cancer (ICC) and two case-control studies of cervical carcinoma in situ (CIS) all carried out by IARC., published before 2000.
Rob et al., 2017	Concordance of HPV-DNA in cervical dysplasia or genital warts in women and their monogamous long-term male partners	J Med Virol 89:1662–70	Overlap with a study from the same research group that was included (Rob et al., 2017 [20])
Grabowski MK et al., 2016	Partner Human Papillomavirus Viral Load and Incident Human Papillomavirus Detection in Heterosexual Couples	J Infect Dis 231:948–56	Unrelated to the study subject
Widdice L et al., 2013	Concordance and transmission of human papillomavirus within heterosexual couples observed over short intervals.	J Infect Dis 207:1286–94	Unrelated to the study subject
Tobian A et al., 2011	Male foreskin and oncogenic human papillomavirus infection in men and their female partners	Future Microbiol 6:739–45	Unrelated to the study subject
Castellsagué X et al., 2002	Male circumcision, penile human papillomavirus infection, and cervical cancer in female partners.	N Engl J Med. 346:1105–12	Combined data collected in five case-control studies of invasive cervical cancer (ICC) and two case-control studies of cervical carcinoma in situ (CIS) all carried out by IARC., published before 2000.
Frega A, 2006	Prevalence of acetowhite areas in male partners of women affected by HPV and squamous intra-epithelial lesions (SIL) and their prognostic significance. A multicenter study	Anticancer Res. 26:3171–4.	Unrelated to the study subject
Gupta A, 2006	Human papillomavirus DNA in urine samples of women with or without cervical cancer and their male partners compared with simultaneously collected cervical/penile smear or biopsy specimens.	J Clin Virol 37:190–4	Unrelated to the study subject
Morales R et al., 2012	HPV in female partners increases risk of incident HPV infection acquisition in heterosexual men in rural central Mexico	Cancer Epidemiol Biomarkers Prev. 21: 956–65	The study group did not include women with CIN
Nicolau SM et al., 2005	Human papillomavirus DNA detection in male sexual partners of women with genital human papillomavirus infection	Urology 65:251–5	The study group did not include male partners of women with CIN

Τhe articles were classified based on the time period when the samples were collected rather than the year of publication (Table 1). Five studies originated from Brazil, two from Spain, one from Netherlands, one from Italy, one from Mexico, one from Colombia and one from Czech Republic. There were differences regarding the time of the samples’ collection: in eight before 2010, in three after 2010. Only one study did not mention the period of the collection of specimens. This study was published in 2012, took the ethical approval in 2009 and the collection of samples would have been rationally done after 2009 and before 2012; so, it was placed between Afonso (collection 2000–2010) and Rob (2013–2015).

In these studies, a variety of molecular assays were used for the detection of HPV in samples, obtained from men, who were sexual partners of women with CIN (Table 3). Information regarding the characterization of HPV subtypes (both HR and LR) were given in nine studies; the remaining three studies assessed HPV detection without subtyping (Table 3). In these three studies the methods used (PCR with universal primers followed by restriction or hybridization) had only the capacity to detect the virus and not to identify subtypes.

**Table 3:** Characteristics of diagnostic approaches for HPV sampling and detection

Name, year	Diagnostic approaches	Sampling methods	Methods of hpv detection	Characterization of HPV sub-types
Bleeker 2005	Peniscopy, HPV DNA Test	Brushes from the top of the penis(glans, corona, sulcus, frenulum, inner part of the foreskin)	HPV GP5+/6+ enzyme immunoassay PCR	HR-HPV: 16, 18, 31, 33, 35, 39, 45, 51, 52, 56, 58, 59, 66, 68. and LR-HPV: 6, 11, 26, 34, 40, 42, 43, 44, 53, 54, 55, 57, 61, 70, 71, 72, 73, 81, 82/MM4, 83, 84, CP6108
Rosenblatt 2004	Peniscopy, Biopsy, HPV DNA Test	Brushes from the penile shaft, the dorsal and ventral prebalanic area, the foreskin and the urethral meatus to navicular fossa	HPV -hybrid capture	HR- HPV: 16, 18, 31, 33, 35, 39, 45, 52, 56, 58, 59, 68 and LR- HPV: 6, 11, 42, 43, 44.
Rombaldi 2006	Peniscopy, Biopsy, HPV DNA Test	Urotest brush from urethral canal, areas identified by peniscopic images as being clinical or subclinical signifance regarding HPV, dorsal and ventral pre-glans region, preputial mucosa, penile shaft	PCR protocol which amplified a 450-bp segment of a conserved region of the L1 viral gene delineated by the MY9 and MY11 primers. For the viral typing: RLFP	N/A
Giraldo 2008	Peniscopy, Biopsy, HPV DNA Test	Brushes from base, body, balanopreputial folds, preputium, distal urethra	Second-generation hybrid capture	HR- HPV: 16, 18, 31, 33, 35, 39, 45, 51, 52, 56, 58, 59, 68
Benevolo 2008	HPV DNA Test	Cytobrush from dorsal and ventral area of the penile shaft, external and internal surface of the prepuce, coronal sulcus, glans and distal urethra	PCR and reverse dot blot hybridization	HR- HPV: 16, 18, 31, 33, 35, 39, 45, 51, 52, 53, 56, 58, 59, 66, 68, 73, 82. and LR-HPV: 6, 11, 40, 43, 44
Guzman-Esquirel 2009	HPV DNA Test	Cytobrush from the surface of the balano-preputial groove, the glans, and with rotating movements the navicular fossa	PCR with HPV universal primers followed by RSA1 endonuclease restriction enzyme	N/A
Martin-Ezquerra 2012	Peniscopy, Cytology and HPV DNA Test	Brushes from the glans, corona, sulcus at baseline and after 6 months. Anal scrapings obtained from anus at baseline. Urine samples obtained at baseline	HCII assay	N/A
Afonso, 2013	Peniscopy, Biopsy, HPV DNA Test	Urotest brush in areas identified by peniscopic images as being of clinical or subclinical significance	HPV detection: PCR using consensus primers MY09/11, HPV genotyping: PCR with primers for the E6 gene DNA sequence of HPV6,11,16, 18, 31,33,35,45,58	HR-HPV:16, 18, 31, 33, 35, 45, 58 and LR- HPV:6, 11,
de Lima Rocha 2012	HPV DNA test	Brushes from the glans and prepuce internal surfaces, including the sulcus and the corona	PCR using GP5+/GP6+ for HPV-DNA detection, followed by PCR using primers specific for 6/11, 16, 18, 31, 33 and 45	HR-HPV:16, 18, 31, 33, 45 and LR- HPV:6, 11,
Rob 2017	HPV DNA Test	FLOQ Swabs brush from the glans of penis, foreskin, urethral orifice, body of the penis and scrotum	PCR with broad spectrum primers and reverse line blot hybridization	HR- HPV: 16, 18, 31, 33, 35, 39, 45, 51, 52, 56, 58, 59, 68, Probably HR- HPV: 26, 53, 66, 67, 70, 73 and LR-HPV: 6, 11, 32, 40, 42, 43, 44, 54, 61, 62, 72, 74, 81, 90
Vargas 2016	HPV DNA Test	Self -obtained penile samples, collected with a sterile nylon cytobrush from the penile groove area, the glans penis, penile body and prepuce	Linear Array HPV Genotyping Test (Roche Diagnostics, Indianapolis, Indiana, USA)	HR-HPV: 16, 18, 31, 33, 35, 39, 45, 51, 52, 56, 58, 59, 66, 68 and LR- HPV:6, 11, 26, 40 42, 53, 54, 55, 61, 62, 64, 67, 69, 70, 71, 72, 73, 81, 82, 83, 84, IS39 and CP6108
Lopez-Diez 2017	HPV DNA Test	Cytobrush from the dorsal and ventral area of the penile, external and internal surface of prepuce, coronal sulcus, glans and distal urethra	Linear Array HPV Genotyping Test (Linear Array, Roche Diagnostics, Mannheim, Germany)	HR- HPV: 16, 18, 26, 31, 33, 35, 39, 45, 51, 52, 53, 56, 58, 59, 66, 67, 68, 69, 70, 73, 82-including IS39 subtype

*N/A* Not available information in study, *HR-HPV* High-Risk HPV, *LR-HPV* Low-Risk HPV

Regarding the collection of specimens, the majority of the studies describe similar anatomical sites (the penile groove area, the glans penis, penile body and procure) for sampling by brushing (Table 3). Only one study used self-obtained samples as previously described by Weaver et al. [23]. Apart from the HPV DNA test, half of the studies used peniscopy as an additional diagnostic tool.

Table 4 describes the characteristics of the couples. Four articles included only monogamous couples, while the remaining eight articles either did not mention whether couples were monogamous or if they included both monogamous and non-monogamous couples. In addition, differences in the time of relationship were also observed; four studies included couples with minimum duration of 6 months, two studies at least 1 year, one study at least 2 years and five studies did not mention the duration of the relationship.

**Table 4:** Characteristics of participants

Name, year	Number of male partners of women with CIN	Mean age	Number of women	Mean age	CIN classification	Clinical symptoms in men	Duration of relationship	*Stable relations*	*Circumcised*	Condom use	Number of sexual partners up to the date of survey
Bleeker 2005	238	37.6y	N/A	N/A	N/A	N/A	N/A	N/A	5%	N/A	N/A
Rosenblatt 2004	30	N/A	30	N/A	CIN I: 15, CIN II: 7 and CIN III: 8	N/A	At least 2 years	Monogamous relationship for at least 2 years	N/A	N/A	N/A
Rombaldi 2006	99	31.7y	N/A	N/A	N/A	N/A	N/A	N/A	8%	40%	50% had 1–10 partners 50% had > 10 partners
Giraldo 2008	54	29y	N/A	N/A	LSIL	Asymptomatic	N/A	N/A	N/A	N/A	N/A
Benevolo 2008	58	37.6y	58	N/A	previous CIN (not longer than 12 months): 31, CIN/ condylomatosis: 27	Asymptomatic	At least 1 year	Monogamous relationship	Exclusion criteria	Exclusion criteria	N/A
Guzman-Esquivel 2009	21	N/A	21	N/A	LSIL	N/A	At least 1 year	Monogamous relationship	N/A	N/A	N/A
Martin-Ezquerra 2012	91	34.3y	N/A	N/A	CIN II or III during the 6 months prior to enrolment	N/A	N/A	N/A	N/A	29%	10 partners
Afonso 2013	60	38.6y	60	34.7y	CIN I: 25, CIN II: 21 and CIN III: 14	N/A	N/A	N/A	N/A	21.1%	N/A
de Lima Rocha 2012	43	N/A	23	N/A	20 LSIL and 3 HSIL	Asymptomatic	At least 6 months	81% Monogamous relationship	7%	14%	N/A
Rob 2017	41	32.4y	N/A	N/A	CIN grade II and III; GW	N/A	At least 6 months	Monogamous relationship	N/A	50%	59.2%: 1–10 partners: 40.8%: > 10 partners:
Vargas 2016	25	36.9y	25	30.6y	ASCUS: 15, LSIL: 8, HSIL:2	N/A	At least 6 months	N/A	N/A	N/A	N/A
Lopez-Diez 2017	125	38.2y	125	35.3y	CIN II: 55 CIN III /CIS: 70	Asymptomatic	At least 1 year	Non- obligatory monogamous relationship	N/A	N/A	23.2% 1–5 partners: 76.8% > 5 partners:

*ASCUS* Atypical Squamous Cells of Undetermined Significance, *CIN* Cervical Intraepithelial Neoplasia, *CD* cervical dysplasia, *CIS* carcinoma in situ*, GW* genital warts, *HSIL* High-grade squamous intraepithelial lesion, *LSIL* Low-grade squamous intraepithelial lesion, *N/A* Not available information in study

Regarding the circumcised participants, four studies included circumcised male partners, one study excluded them; the rest of the studies did not mention if their male partners were or were not circumcised. On the other hand, differences in the use of condoms have also been observed; six studies mentioned the percentage of participants which used condoms, one excluded couples who used condoms and the remaining five articles did not mention the percentage of condom use. Finally, only five studies described the proportion of women with CIN I/ II/ III, whose partners participated in the studies.

The number of men who were partners of women with CIN, was 885. The mean age of the male participants was 35.18 years and the standard deviation was 3.47. The coefficient of variation was 9.8%. A total of 779 penile samples were obtained by brushing, and were tested by any HPV molecular assay; among them, 383 (49.1%) were HPV-positive. Ten out twelve studies (83.3%) demonstrated prevalence > 20%. A great difference regarding the HPV prevalence was observed between the studies, depending on the particular profile of the target group, on the method assay used and on the prevalence of the virus in the various geographical areas (Table 5). The lowest percentage (12.9%) was found by Martin-Ezquerra et al in Spain [17], whereas, the highest (86%) was described by de Rocha et al in Brazil [19].

**Table 5:** Results of Studies

*Name, year*	Positive peniscopy in partners of women with CIN	HPV DNA by brushing in partners with CIN %	HPV DNA test from urine	HPV DNA test from biopsy	Most other frequent detected subtype subtypes	Number of samples positive for HR vs LR-HPV
Bleeker 2005	139/238 (58.4%)	101/170^b^ (59.4%)	N/A	N/A	HPV 16 6,31,33,18	81/101 (80.2%) HR vs 32/101 (31.6%) LR^a^
Rosenblatt 2004	5/30 (16.7%)	7/30 (23%)	N/A	3/30 (10%)	N/A	3/7 (42.8%) HR vs 4/7 (57.2%) LR
Rombaldi 2006	62/99 (62.6%)	54/99 (54.5%)	N/A	N/A	HPV 6 11,16,40,61,84	2/54 (3.7%) HR vs 52/54 (96.3%) LR
Giraldo 2008	13/54 (24%)	14/54 (25.9%)	N/A	N/A	N/A	Only HR subtypes tested
Benevolo 2008	N/A	25/54^b^ (46.2%)	N/A	N/A	HPV 16 51,52,53,56,58,59 31,33,34,35,39,66, 68,73,82,6,11,40,43,44	22/25 (88%) HR vs 3/25 (12%) LR
Guzman-Esquivel 2009	N/A	4/21 (19%)	N/A	N/A	N/A	1/4 (25%) HR vs 3/4 (75%) LR
Martin-Ezquerra 2012	11/91 (12%)	8/62^b^ (12.9%)	22/78^b^ (28%)	N/A	N/A	N/A
Afonso 2013	22/60 (36.7%)	30/60 (50%)	N/A	N/A	HPV 16 45,18	18/30 (60%) HR vs 15/30 (50%) LR^a^
de Lima Rocha 2012	N/A	37/43 (86%)	N/A	N/A	HPV 16 6,11,31,18,33,45	29/37 (78.3%) HR vs 23/37 (62.1%) LR^a^
Rob 2017	N/A	26/36^b^ (72.2%)	Ν/Α	Ν/Α	HPV 16 6,11,18,30,31,33, 35,39,40,42,51,52, 53,54,56,58,59,68, 70,73,74,81,82,90	23/26 (88.4%) HR vs 8/26 (30.7%) LR^a^
Vargas 2016	N/A	14/25 (56%)	N/A	N/A	HPV 83 16,62, 68,81,59,51, 31, 45,6108,34,82, 73,71,67,54,53,52	11/14 (78.5%) HR vs 10/14 (71.4%) LR^a^
Lopez-Diez 2017	N/A	63/125 (50.4%)	N/A	N/A	HPV 16 18, 33,52,51,31,39 45,56,58,59,53,66, 67,68,69,70,73	N/A

*N/A* Not available information in study, *HR-HPV* High-Risk HPV, *LR-HPV* Low-Risk HPV

^a^In these studies, there have been some specimens with both High and Low-Risk HPV subtypes

^b^Unsuccessful PCR analysis in some specimens in the selected studies

In addition, among the studies, different HPV subtypes were identified, such as, 6, 11, 16, 18, 31, 33, 40, 42, 45, 51, 52, 53, 54, 56, 57, 58, 59, 61, 62, 66, 68, 81 and 83 (see Table 5). According to the data of six studies, HPV 16 was the most frequent subtype [11, 15, 18–20, 22], whereas, in two other studies the subtypes 6 and 83 predominated [13, 21].

Finally, only five out of 12 studies have studied the concordance of HPV-subtyping between the couples [15, 17, 19, 21, 22]. Benevolo et al*,* reported that 42.8% of the couples, that were HPV-positive, harbored at least one identical subtype, including HPV 16, 51, 52, 53, 56 and 58 [15]. This percentage is lower than that described by Lopez-Diez et al*,* who has demonstrated that 62% of infected couples had at least one subtype in common [22]. In both studies, HPV 16 was detected in a high proportion of infected couples. Vargas et al also showed that 28% of the sexual partners shared at least one viral subtype (16, 51, 52, 54, 68, 73 and 81) [21], while Afonso et al demonstrated that 53.3% of the couples had the same subtype (HPV 16) [18]. de Lima-Rocha et al found that 56.5% of the couples had at least one common subtype (HPV 6, 11, 16, 18, 31), whereas, absolute concordance was observed in only one case (4.3%) [19]. Furthermore, in the same study, the male partner had the same HR sub-types as their female sexual partner (HPV 16, 18, 31).

## Discussion

HPV infection is common in asymptomatic men. In a systematic review of the literature, Dunne et al have shown that the prevalence of HPV infection in asymptomatic men ranges from 1.3–72.9% [24], while Smith et al have demonstrated that HPV prevalence among high-risk men (such as sexually transmitted infection clinic attendees, human immunodeficiency virus-positive males, male partners of women with HPV infection or abnormal cytology and men who have sex with men) was from 2 to 93% versus 1–84% in low-risk men [25–27]. In addition, the Centers of Disease Control and Prevention (CDC) reported that, between 2013 and 2014, in the USA, the prevalence of genital HPV for men aged from 18 to 59 years old was 45%, while, 25% of men had HR genital HPV infection [28]. A recent meta-analysis revealed a prevalence of 49% of any type of HPV and 35% of HR-subtypes in men [29]. However, the real incidence and prevalence of HPV infection in asymptomatic men is difficult to estimate, due mainly to the silent behavior of this virus not only in men but also in women.

According to data from studies conducted in North and Latin America, genital HPV prevalence is indicated to be higher in men than in women [30–32]. A meta-analysis by de Sanjose et al has demonstrated that the overall HPV prevalence in women with normal cervical cytology was 10.4%; the highest percentages were observed in Africa (22.1%), Central America and Mexico (20.4%), Northern America (11.3%), Europe (8.1%), and Asia (8.0%) [33]. On the basis of these estimates, approximately 291 million women worldwide are carriers of HPV DNA, of whom 32% are infected with HPV 16 or 18, or both.

In the present systematic review, we have found that the mean prevalence of HPV infection, in male partners of women with CIN was 49.1%. Although HPV 16 was the most common subtype, many other subtypes (6, 11, 16, 18, 31, 33, 40, 42, 45, 51, 52, 53, 54, 56, 57, 58, 59, 61, 62, 66, 68, 81 and 83) were also detected. An interesting finding was that none of the participants had any clinical signs indicating that this target group was a reservoir for the dissemination of the virus. Apart from the HPV detection, the subtyping is very important not only for epidemiological purposes but, also, for evaluating the oncogenic potential of new subtypes in order to establish an effective and safe vaccination program.

Despite the variety of methods for the diagnosis of HPV in men, investigation into the presence of the virus has not been consensual [34]. Unfortunately, the identification of the presence of HPV in men is far more difficult than in women, due to the smaller quantity of plane squamous non-keratinized mucosa of the male genital organ in relation to that of the female [35]. Diagnostic tools are peniscopy, biopsy and HPV DNA testing. Recent studies have demonstrated that even when carried out by experienced professionals, peniscopy has very low specificity, leading to unnecessary biopsies [36]. Today, the identification of HPV has been carried by molecular assays using polymerase chain reaction (PCR) or hybrid capture; these methods are rapid, sensitive and easy to be performed [37–40]. Several of them are commercial, having the capacity not only to detect infection presence but also to characterize the different subtypes. Clinical specimens obtained using brushes from different anatomical sites can be directly tested for the presence of HPV DNA. Giuliano et al have shown that the optimal anatomical sites for detection of HPV are the penile shaft, the glans penis/coronal sulcus and the scrotum; specimens obtained from urethra and semen seem to have the lowest sensitivity [32]. However, sometimes, the detection of genital HPV is technically more complicated in men than in women because cells are more difficult to harvest from skin than from moist mucosal surface. Specimens, such as urine, are more easy to be obtained and to be tested for HPV presence. Neha Pathak et al*,* have demonstrated that HPV DNA testing of urine may be an alternative and an easier approach [41, 42].

According to the data of the studies included in this review, the use of condoms was limited. In addition, only a minority of participants were circumcised. Previous studies have demonstrated that constant condom use is associated with reduced prevalence of HPV [43]. On the other hand, circumcision seems to minimize the risk for HPV penile infection and in the case of men with multiple sexual partners, circumcision reduce the risk of cervical cancer in their sexual partners [44].

There is a question how to manage the male partners of women diagnosed with CIN. It is well known than men who are found positive for HPV, could be HPV negative after 12 months. This could be explained by the fact that the epithelial cells of the penile skin are more resistant to HPV infection than the cervical epithelium, the clearance rates differ by gender and the duration of HPV infection is shorter in men than in women [45]. Morales et al have shown that the median clearance time for any HPV subtype was 5.1 months (3.5–7.7), while the duration of the colonization was similar for oncogenic and nononcogenic HPV subtypes [46]. Also, Guiliano et al stated that the median clearance rate of any HPV subtype was 5.9 months, with no observed difference in clearance time between oncogenic and nononcogenic HPV subtypes; 75% of participants were negative for any HPV subtype after 12 months [47].

Summarizing the results of the studies included, healthy sexual male partners of women with CIN may be HR HPV-positive, maintaining the risk of viral transmission and consequently the risk of recontamination of their female partners. Therefore, when the male partners were found to be positive by a penile HPV test, they should be advised to undergo a clinical follow up as previously reported by Gupta et al [48]. In addition, the introduction of the 9-valent HPV vaccine, that includes the subtypes 6, 11, 16, 18, 31, 33, 45, 52 and 58, most of which were detected in the partners of women with CIN, combined with education regarding the prevention should limit the spread of the virus within couples [49, 50].

## Limitations

The main limitations of this review are the differences between the characteristics among the participants and the different HPV DNA testing assays. Some studies have included regular and monogamous sexual partners, but others just regular partners. Some studies have included men who have been circumcised, which may alter the results since circumcision seems to protect men from HPV infection. Among the studies, the proportion of women with CIN I, CIN II and CIN III differed significantly, while sometimes the proportion was not mentioned. So, searching for correlation between HPV infection of male partner and the grade of CIN was not possible. Also, the studies have been held in different countries, where the prevalence of HPV infection varies in the general population. Finally, among the studies, various HPV DNA tests with different specificity were used for the detection of the virus, in addition, each study characterized specific subtypes.

## Conclusion

Until now, there are not precise screening or surveillance guidelines for the management of partners of women with CIN. This population is frequently colonized by various HPV subtypes and therefore need to be screened in an effort to reduce the infection in both sexes. The screening test could include detection/identification of HPV subtypes by a molecular assay, followed by peniscopy only in the positive cases.

Given that the virus is associated with neoplastic lesions, these men are also at risk for HPV-related tumors (penile cancer etc). The introduction of vaccines could play an important role to the prevention and therapy. Prophylactic HPV vaccination (B-cell-mediated immunity) provides lifelong protection against subtypes included in the vaccine; therefore, a vaccination program for children of both sexes is counted to the primary prevention strategies and might reduce the HPV prevalence. On the other hand, therapeutic vaccines, based on an antigen-specific T-cell immunity are promising approaches for the treatment of already existing intracellular HPV infections and are under investigation.

## Abbreviations

ASCUS: Atypical Squamous Cells of Undetermined Significance
CD: Cervical dysplasia
CDC: Center for Control and Prevention
CIN: Cervical Intraepithelial Neoplasia
GW: Genital warts
HPV: Human Papillomavirus
HR: High risk
HSIL: High-grade squamous intraepithelial lesion
LR: Low-risk
LSIL: Low-grade squamous intraepithelial lesion
N/A: Not available information in study
